# Editorial for the Special Issue “Effects of COVID-19 on Lifestyle Behaviors in Children with Obesity”

**DOI:** 10.3390/nu15122652

**Published:** 2023-06-07

**Authors:** Anna Ferrulli, Ileana Terruzzi, Gianvincenzo Zuccotti, Livio Luzi

**Affiliations:** 1 Department of Endocrinology, Nutrition and Metabolic Diseases, IRCCS MultiMedica, 20138 Milan, Italy; ileana.terruzzi@unimi.it (I.T.); livio.luzi@unimi.it (L.L.); 2 Department of Biomedical Sciences for Health, University of Milan, 20133 Milan, Italy; 3 Department of Biomedical and Clinical Sciences, University of Milan, 20157 Milan, Italy; gianvincenzo.zuccotti@unimi.it; 4 Department of Pediatrics, Buzzi Children’s Hospital, 20157 Milan, Italy

During the last four decades, the prevalence of obesity has increased dramatically worldwide; concomitantly, a progressive rise in the prevalence of obesity, diabetes, and other nutrition-related chronic diseases has also been observed in childhood [1]. From the end of 2019/beginning of 2020, another serious pandemic, caused by the SARS-CoV-2 virus, has affected, and disrupted the lives of the world’s population. A complex and bi-directional relationship between the pandemics of obesity and COVID-19 exists. According to several studies, adjusted for age, gender, social class, diabetes, and heart conditions, an elevated Body Mass Index [BMI] has been shown to be a strong independent risk factor for severe COVID-19 infection and for increased SARS-CoV-2-related mortality, both in adults [2] and in children [3]. Several pathophysiological mechanisms are hypothesized to underlie the interrelationship between pediatric obesity and severe SARS-CoV-2 infection; notably, an impairment of a correct immune system functioning has been observed in children with obesity, induced by hyperglycemia, hyper-insulinemia, dyslipidemia, proinflammatory state, and respiratory and cardiovascular problems associated with endothelium damage [4]. Furthermore, nutritional deficiencies, such as vitamin D deficit, and, less commonly, vitamin B12, C, A, E, iron, and folate deficiencies, as well as an altered gut microbiota composition (unbalanced *Firmicutes*/*Bacteroidetes* ratio), both probably due to an incorrect feeding (low consumption of fruits and vegetables and increased “junk” food consumption), contribute to increased susceptibility to SARS-CoV-2 infection in children with obesity [4].

Conversely, the COVID-19 pandemic severely impacted body weight and the general health of the worldwide population, caused by changes in lifestyle habits, physical inactivity, and social isolation, in turn due to lockdowns and “stay-at-home” instructions. Specifically, children and adolescents, although less directly affected by SARS-CoV-2, have paid a heavy price through the indirect effects of the crisis, including unregulated feeding, leading to an increased risk of both obesity and being underweight, a sedentary lifestyle, mental health impacts and social isolation, addiction to screens, and a lack of schooling and healthcare [5].

The purpose of this Special Issue was to collect original articles or reviews aimed at exploring lifestyle behavioral changes during lockdown periods in children and adolescents, as well as their impact on body weight. Furthermore, potential mechanisms underlying the impact of SARS-CoV-2 on body weight have been investigated, such as the involvement of innate and adaptive immunity, or COVID-19-induced smell and taste changes or increased emotional eating. The pandemic has also favored the spread of new technologies, such as telemedicine, for the follow-up of pediatric patients with obesity during the lockdown, ushering in a new era of chronic patient care at home.

Several studies evaluated the effects of the first COVID-19 lockdown on the eating habits of children and adolescents. Specifically, an Italian cross-sectional survey involved a total of 439 participants, differentiated in children (five to nine years) and adolescents (ten to fourteen years) from northern and southern Italy. Participants were asked, via an online questionnaire whether, during the COVID-19 quarantine, is there was an increase or reduction in the intake of selected foods, as well as in soft drink consumption and in body weight [6]. Children and adolescents were found to increase consumption of “comfort food” (e.g., chocolate, packaged sweet snacks, ice cream and desserts, as well as pasta, rice, bread, pizza, and bakery products). In about 60% of the enrolled population, the change in eating habits was associated with an increase in body weight (about more than 3 kg), significantly more frequent in adolescents than in children (67% vs. 55%), with some differences also regarding the type of most consumed foods [6]. A comparable result in terms of increase in body weight (about 2.67 kg) and BMI (0.77 kg/m^2^) in school-age children, during the COVID-19 confinement period, has been reported by a meta-analysis, which analyzed 12 articles; the prevalence of pediatric obesity and being overweight also increased compared to before the pandemic. Once again, the main factors contributing to body weight gain in children during the pandemic were prolonged periods of sedentary behavior, decreased physical activity, and changes in dietary habits [7].

In addition to changes in lifestyle habits, one of the possible mechanisms that are hypothesized to contribute to weight gain during the COVID 19 pandemic is emotional eating, resulting from less effective coping skills in response to negative emotions and stressful events. Concerning this, a study involving 1126 Polish adolescents (aged 15–20) investigated the association between emotional eating (assessed through the Emotional Eater Questionnaire (EEQ)) and BMI during the COVID-19 pandemic period [8]. A higher rate of emotional eaters/very emotional eaters and a more frequent number of emotional eating behaviors emerged among participants that were overweight and obese compared to the other groups [8]. The female gender showed a greater predisposition to emotional eating behaviors, arguably due to hormonal and genetic causes, which supported the data in the scientific literature [9,10].

Besides the psychological and emotional aspects, numerous studies agree in stating that, also, altered chemosensory perception (hyposmia/dysosmia and of hypogeusia/dysgeusia) induced by SARS-CoV-2 infection has been responsible for behavioral changes in food intake during the COVID-19 pandemic, contributing to variations in BMI in the general population, as well as among children and adolescents [11,12]. Most studies on this topic argue that the altered chemosensory perception (taste and smell) mainly induces reduced appetite, leading to a faster fullness sensation during the consumption of a meal and, therefore, to a decrease in body weight. On the other hand, a reduced perception of a food’s sensory properties may trigger compensatory responses that lead some individuals to increase food intake with an opposite effect on body weight [11].

As mentioned previously, the intricate inter-relationship between COVID-19, immune function, obesity-related inflammation, and inadequate nutrient intake is also thought to be responsible for the increased severity of COVID-19 infection in both adults and children/adolescents with obesity [13]. The exact pathogenetic mechanisms underlying these effects are not fully understood. However, the adipose tissue is thought to act as a SARS-CoV-2 reservoir due to its high expression of ACE2, the functional receptor for SARS-CoV-2; moreover, the excess of adipose tissue causes an imbalance between anti-inflammatory and pro-inflammatory cells/cytokines, in favor of the latter, leading to a chronic mild-grade systemic inflammation, as shown by circulating levels of cytokines and acute phase proteins [13]. In turn, this mild-grade inflammation impairs adipocyte function and immune state dysregulation in individuals with obesity compared to normal weight individuals, raising the risk of more severe infections in obesity. Additionally, nutritional deficiencies frequently observed in individuals with obesity may weaken immune function, establishing a vicious circle [13].

Concerning the category of children/adolescents that are overweight or obese, containment measures adopted during the COVID-19 pandemic significantly impacted other childhood nutrition and metabolic diseases [14]. During the COVID-19 pandemic, an increased number of new diagnoses of Type 1 Diabetes (T1D) has been reported in children [15]. Although a certain common immunological mechanism has not yet been demonstrated, it is now established that changes in the availability of healthcare services and limited accessibility to healthcare services led to delayed diagnosis and more severe presentation of T1D, such as diabetic ketoacidosis [14]. Furthermore, several studies agree that, during the COVID-19 pandemic, there has also been a significant increase in the onset rate of Type 2 Diabetes (T2D) and delayed referral to hospitals compared to previous years.

The detrimental impact of the COVID-19 pandemic on physical and psychological public health, involving child and youth population groups, has amplified the need for strategies to contain the nutritional and metabolic impact of new possible future pandemics.

Physical activity and exercise are effective tools to counteract body weight increase in pediatric and adolescent populations, especially if it is associated with changes in eating habits [16]. Furthermore, it is well known that healthy lifestyles and behaviors potentiate the immune system, reducing the risk for infections and inflammation; specifically, regular physical activity is associated with the reversal of systemic inflammatory conditions seen in subjects presenting as overweight and obese, characterized by the release of proinflammatory cytokines and hormonal dysregulation [16]. In order to encourage the practice of physical activity, despite the restrictions during the lockdown period, some authors proposed alternative types of home-based setting exercises, such as exergaming, constituted of specific videogames and devices that copy the body movements commuting into the avatar’s moving on the screen or telehealth physical activity programs for assisting and monitoring children that are overweight and obese during their remote exercise programs [16].

The spread of telehealth technology during the COVID-19 pandemic was revealed to be useful for ensuring adequate healthcare services by health care professionals in different fields. The application of telehealth was particularly effective for monitoring whole health and nutritional status in children and adolescents presenting as overweight/obese by tracking any comorbidities associated with obesity, ensuring continuity in dietary intervention, providing information to the family while respecting the specifics of the condition, supervising programs of physical activity, providing psychological support, and increasing patient satisfaction [17].

Despite the COVID-19 pandemic having caused a great global health crisis of our time, it has been useful in raising issues related to the management of certain categories of patients and in providing lessons for handling possible future pandemics. In the context of childhood and youth obesity, the COVID-19 pandemic has taught us that it is essential to identify the society groups of more vulnerable subjects with increased risk of developing complications, such as children or adolescents already suffering from being overweight and being obese (mainly of the female gender), or with less effective coping skills, in response to negative emotions and stressful events, leading to “emotional eating”. Education regarding a balanced diet and regular physical activity is essential, beginning from childhood, and it can be encouraged using new attractive technologies, such as exergaming or telehealth physical activity programs. The importance of an adequate supply of nutrients and regular physical exercise during childhood and adolescence is also linked to the ability to strengthen the immune system (also through a normalization of the composition of the intestinal microbiota) and, therefore, to counteract viral infections. Finally, the COVID-19 pandemic had the merit of promoting the spread and the use of telehealth in the management and follow-up of chronic diseases. Telemedicine offers innovative access to health care, ensuring the supportive and comprehensive care of children and their families, as well as guaranteeing a systematic assessment of their health and biopsychosocial needs, which is critical for reducing the negative impact of obesity and other possible future pandemics [16,18].
